# Co-producing principles to guide health research: an illustrative case study from an eating disorder research clinic

**DOI:** 10.1186/s40900-023-00460-3

**Published:** 2023-09-20

**Authors:** Cat Papastavrou Brooks, Eshika Kafle, Natali Butt, Dave Chawner, Anna Day, Chloë Elsby-Pearson, Emily Elson, John Hammond, Penny Herbert, Catherine L. Jenkins, Zach Johnson, Sarah Helen Keith-Roach, Eirini Papasileka, Stella Reeves, Natasha Stewart, Nicola Gilbert, Helen Startup

**Affiliations:** 1 Population Health Sciences, Bristol Medical School, University of Bristol, Canynge Hall, 39 Whatley Road, Bristol, BS8 2PS UK; 2 SPIRED Clinic, Research and Development Department, Sussex Partnership NHS Foundation Trust, Sussex Education Centre, Nevill Avenue, Hove, BN3 7HZ UK; 3 Comedy for Coping, Aesthetics Research Centre, University of Kent, Room 2.16, Jarman Building, Canterbury, Kent, CT2 7UG UK; 4 Department of Psychology, City, University of London, Northampton Square, London, EC1V 0HB UK; 5 School of Human and Behavioural Sciences, Bangor University, Bangor, LL57 2DG UK; 6 Maudsley Learning, ORTUS Conferencing and Events Venue, 82-96 Grove Lane, London, SE5 8SN UK

**Keywords:** Co-production, Lived experience, Eating disorders, PPI, Research priority setting, Service-user led research, Anorexia nervosa, Bulimia nervosa

## Abstract

**Background:**

There is significant value in co-produced health research, however power-imbalances within research teams can pose a barrier to people with lived experience of an illness determining the direction of research in that area. This is especially true in eating disorder research, where the inclusion of co-production approaches lags other research areas. Appealing to principles or values can serve to ground collaborative working. Despite this, there has not been any prior attempt to co-produce principles to guide the work of a research group and serve as a basis for developing future projects.

**Methods:**

The aim of this piece of work was to co-produce a set of principles to guide the conduct of research within our lived experience led research clinic, and to offer an illustrative case for the value of this as a novel co-production methodology. A lived experience panel were recruited to our eating disorder research group. Through an iterative series of workshops with the members of our research clinic (composed of a lived experience panel, clinicians, and researchers) we developed a set of principles which we agreed were important in ensuring both the direction of our research, and the way in which we wanted to work together.

**Results:**

Six key principles were developed using this process. They were that research should aim to be: 1) real world—offering a clear and concrete benefit to people with eating disorders, 2) tailored—suitable for marginalised groups and people with atypical diagnoses, 3) hopeful—ensuring that hope for recovery was centred in treatment, 4) experiential—privileging the ‘voice’ of people with eating disorders, 5) broad—encompassing non-standard therapeutic treatments and 6) democratic—co-produced by people with lived experience of eating disorders.

**Conclusions:**

We reflect on some of the positives as well as limitations of the process, highlighting the importance of adequate funding for longer-term co-production approaches to be taken, and issues around ensuring representation of minority groups. We hope that other health research groups will see the value in co-producing principles to guide research in their own fields, and will adapt, develop, and refine this novel methodology.

## Abstract (plain language summary)

It important that when researchers are trying to understand illnesses they do this together with people who have experienced them. This can be difficult, because researchers often take over—even if everyone is meant to be working as a team. We are a group of people trying to understand eating disorders and help people who have them get better. In our group there are some people that have experienced an eating disorder, health workers and researchers.

We thought it might be helpful if we could start by working out what things were most important to us as a group, and then try to stick by them. We talked a lot together to come up with a list of principles.

The six principles we thought were the most important were that research should make a difference to people’s lives, see people as individuals, be hopeful, make sure that people have a voice, look at things that aren’t traditional therapies, and always work together as equals.

There are some issues with what we did; we found it hard to get a good mix of people in our group, and we were lucky in having enough money to pay people to do what we wanted to do, which is not always true. Despite this, we still hope that other teams might look at what we have done, and see if they could build on it, or change it, so it would work for them.

## Background

### Co-production approaches

Co-production has been defined as the delivery of “public services in an equal and reciprocal relationship between professionals, people using services, their families and their neighbours” [21], placing a strong emphasis on the importance of citizens’ power and worth [26]. The initial emergence of co-production within health services stemmed from a critique of the limited power patients had to shape the service they received, and the failure of services to acknowledge and respond to patients’ often negative experiences [32]. Despite the argued importance of co-production in terms of its substantive, instrumental, normative and political value [94] there have been significant criticisms of what is typically described as ‘co-production’ in health services. This includes the claim that co-production rarely involves a transfer of power to patients [136], instead involving a co-option of people’s lived experience to be used for agendas which are foreign to them and their interests [102]. Genuine co-production is often impossible due to the reality of the power imbalances present in the psychiatric system [97], and—by masking and de-politicising these imbalances—it can serve to exacerbate them [124].

Within health research, co-production approaches come with their own associated set of difficulties [79]. Relationships within research often mirror those between patient and clinician and there can be a division between researchers who are seen as active, knowing and rational in contrast to the passive, known and irrational objects of health research—patients [66]. Although evidence-based practice is often reified as a values-neutral methodology [14], it involves the privileging a specific scientific or rationalistic voice, excluding ways of knowing which do not conform to this and often constituting a form of injustice against epistemically marginalized groups [30, 37]. This critique is foundational to the emerging field of mad studies, which seeks not to just include mad people within mental health research by forcing them to assimilate within traditional methodological frameworks but demands instead substantial changes to the way we conceive of and conduct research [44, 63]. Thus the aims of mad studies include both “showing that there is method in our madness; and on the other side, preserving madness in our method” [63].

In addition to these theoretical issues, co-producing research with patients can often have significant psychological and emotional costs for them, partially as a result of increased interpersonal conflict which often occurs [94]. Collaborating with patients on mental health research has been conceptualised as occurring on one of three distinct levels: consultation, collaboration, and user-controlled,Rose and Kalathil [105] argue that consultation is superficial and lacks value, and that collaboration only requires power imbalances to be hidden as opposed to abolished, meaning it inevitably collapses into consultation. On this model, methodologies like participatory action research—which centre around collaboration between researchers and participants [54] though do not challenge either the conceptual distinction or the power-relations between them typically fall under the category of collaboration. Only user-controlled research can preserve patients’ autonomy and ability to affect the research process without the risk of co-option [105].

### Co-producing eating disorder research

Eating disorders (EDs) are a classification of disorders which involve feeding and eating disturbances which cause significant clinical distress [76]. An estimated 725,000 people in the UK are affected by EDs, at an estimated cost of 3.9–4.6 billion pounds annually to the NHS [15]. Anorexia Nervosa (AN) and Bulimia Nervosa (BN) are two eating disorders with high mortality rates, compared to the general population [11, 128] with AN leading to the highest mortality rates compared to all other psychiatric illnesses [11].

Despite this, the strength of evidence for eating disorder treatments is modest [23, 58] with no gold standard psychological interventions available for adult presentations of AN [83] or BN [96]. Systematic reviews of randomised control trials (RCTs) in eating disorders have found the evidence for psychological therapies for eating disorders inconclusive [24, 58]. Therapies for eating disorders have a minimal evidence base, consisting of a very small number of studies [113], with and high impact factor journals publish significantly fewer papers on eating disorders than other psychiatric conditions [113]. This may be due, in part, to a deficit in funding for eating disorder research in comparison to the burden of illness [38, 83], mirroring a lack of funding for clinical services [6].

However, another reason for the lack of effective evidence-based treatments for people with eating disorders could be comparative failings of integrating lived-experience perspectives into research in this area. Aspects of co-production have been incorporated into case-studies [4, 5, 19], the development of novel pathways such as FREED [7], within service delivery [75] and in research priority setting [9, 92]. Feminist approaches within an eating disorder context have also been significant in challenging the politics and validity of diagnoses and power-imbalances within treatment, through drawing on women’s embodied and socially situated lived experience of having an eating disorder [72]. However, co-production within eating disorder research is not as established as service-user /survivor led research in other areas [44, 104], where research teams and projects are either headed by or predominantly composed of lived-experience researchers.

### Principles for co-production

A barrier to co-production in health research more generally, and eating disorder research specifically, might be that people with lived experience of an illness are often brought in to work on a specific research project, the parameters, aims and methodology of which have been set prior to their involvement. This contrasts with longer term work conducted by research teams including people with lived experience as key members—who might have the capacity to work together over a longer time-period to decide the direction they want their research to go in, and co-produce projects from the ground-up. Developing projects according to research principles has been found to be helpful in enabling this kind of longer-term collaboration, particularly around ideas of sharing power and building trust [31]. Many historically significant groups of mental patients advocating for their rights and to change mental health services have manifestos structured around shared values they collectively organize on the basis of CAPO [28], Mental Patients Union [81], MPU [82]. Within mental health services, there has been work carried on co-producing principles to guide the development and evaluation of peer worker roles [50], for best practice in school mental health [133] and within health research to develop principles for community-based research [107]. However, to our knowledge, there has not been an attempt to develop principles to form the basis for co-producing research between people with lived experience of a particular mental health issue and researchers, who though they might happen to have lived experience of what they are researching, are not employed in that capacity. Principles may help to guide the research process, facilitate collaborative working, and form the shared basis from which to derive research priorities. We believe that doing so could be an important way of preventing the collapse of collaboration into consultation [105].

### The SPIRED research clinic

SPIRED (Sussex Partnership Innovation and Research in Eating Disorders) research clinic was founded in January 2021 and is composed of people with lived experience of eating disorders, researchers and clinicians working in the Sussex Eating Disorders Services within Sussex Partnership NHS Foundation Trust (SPFT). These identities are not mutually exclusive; many SPIRED clinic members will fall under two or more of these categories, and we aspire to be a team where clinicians and researchers are able to share their own experiences of mental health issues, regardless of their formal role [70].

To ensure those with lived experience had oversight in the research activities of the clinic, a lived experience panel was recruited in collaboration with SPFT’s Patient and Public Involvement (PPI) team [115]. The PPI team oversees the development of lived experience advisory panels (LEAPs) to ensure that those with lived experience can provide insight into the research conducted within SPFT. An advert was circulated on social media, within SPFT and with partner organisations to recruit individuals with diverse experiences of eating disorders and eating disorder treatment and was also circulated to patients within the trust eating disorder service who were at point of discharge. Sixteen individuals were recruited to the lived experience panel, and consequently SPIRED. The criteria for recruitment were that they had to identify as having experienced an eating disorder, regardless of whether that had been formally diagnosed. They were not required to reveal any information about their eating disorder. Since the eating disorder service SPIRED is connected to is for adults, all SPIRED lived experience panel members are over eighteen. Although these individuals were specifically recruited for a lived experience role, some of them also had clinical experience within the NHS, as well as research expertise. A co-production approach is followed in research conducted by SPIRED [32], echoing the rhetoric 'no decision about me without me' [41]. Within SPIRED, those with lived experience of EDs contribute to developing ideas, overseeing projects and conducting research.

## Methods

### Aims

1. To co-produce a set of principles to guide research, within an eating disorder research clinic composed of people with lived experience of an eating disorder, clinicians, and other researchers.
2. To provide an illustrative case for our approach as a novel co-production methodology, by demonstrating how co-producing developing principles can lead to a clear and defined research agenda.

### Development of principles

Prior to the first SPIRED team meeting, the lived experience panel and SPIRED team members were invited to complete a questionnaire. The questionnaire was developed by the SPIRED clinic research assistant, a Service User and Carer Involvement Coordinator from the SPFT PPI team, and a member of the lived experience panel.

The questionnaire consisted of four open ended questions, and asked members to share whatever they felt comfortable sharing: their experience of eating disorders and eating disorder services, their view of what lived experience involvement in research meant, what they wanted to contribute to SPIRED aside from their lived experience of an eating disorder, and what they felt should be the key priorities for eating disorder research.

Responses were loosely summarised by theme and presented at the first SPIRED clinic meeting (facilitated over an online platform), where seventeen members were present, including eleven members of the lived experience panel. Small group discussions were held in break-out rooms to develop key principles, followed by feedback and a discussion with the whole group. Extensive notes were taken of all these discussions.

These notes formed the starting point of a smaller working group, with three lived experience panel members and two other SPIRED clinic members. A consensus-based decision making methodology [56] was used to derive core principles from the discussions of the wider SPIRED group. All the key principles derived from the initial meeting were discussed individually, with each working group member holding veto-power for any decision around a particular principle (whether that was to include or to exclude it). This then halted the decision, and the principle was then re-discussed and modified until consensus could be reached [56]. Following agreement about which principles to include, this consensus-based process was used for decision-making around which of the principles could be synthesized.

A group facilitator chaired the meeting and made notes on ongoing discussions to enable further reflection. The lead author then wrote up the main sections of this paper, which were then circulated to all SPIRED clinic members for comments and feedback, following which extensive changes were made to this paper.

### Reflexivity

Everyone who has contributed to this paper at any stage has been invited to co-author (including the lived experience panel). Although we felt it was vital to highlight the extent of the contribution of the lived experience panel in the co-production of these principles, we did not want to force any individual to identify themselves as someone with lived experience of an eating disorder. Therefore, all authors are listed solely as SPIRED clinic members.

The SPIRED clinic consists of individuals with a range of experiences of different eating disorders, eating disorder services, clinicians, and researchers. We aimed to ensure representation from neurodivergent people, LGBTIA people, men, and people of colour (POC), all of which are often marginalised within eating disorder treatment. However, we recognise that POC are particularly underrepresented on our lived experience panel, which may have impacted on the principles that our research team chose to prioritise.

### Ethics

The SPFT Research Governance department confirmed that ethical approval was not necessary for this project, as there were no participants.

Because of the increased sensitivity of sharing personal information about mental health, co-authors who were lived experience panel members also signed a consent form to be anonymously quoted in this paper.

## Results

**Fig. 1:**
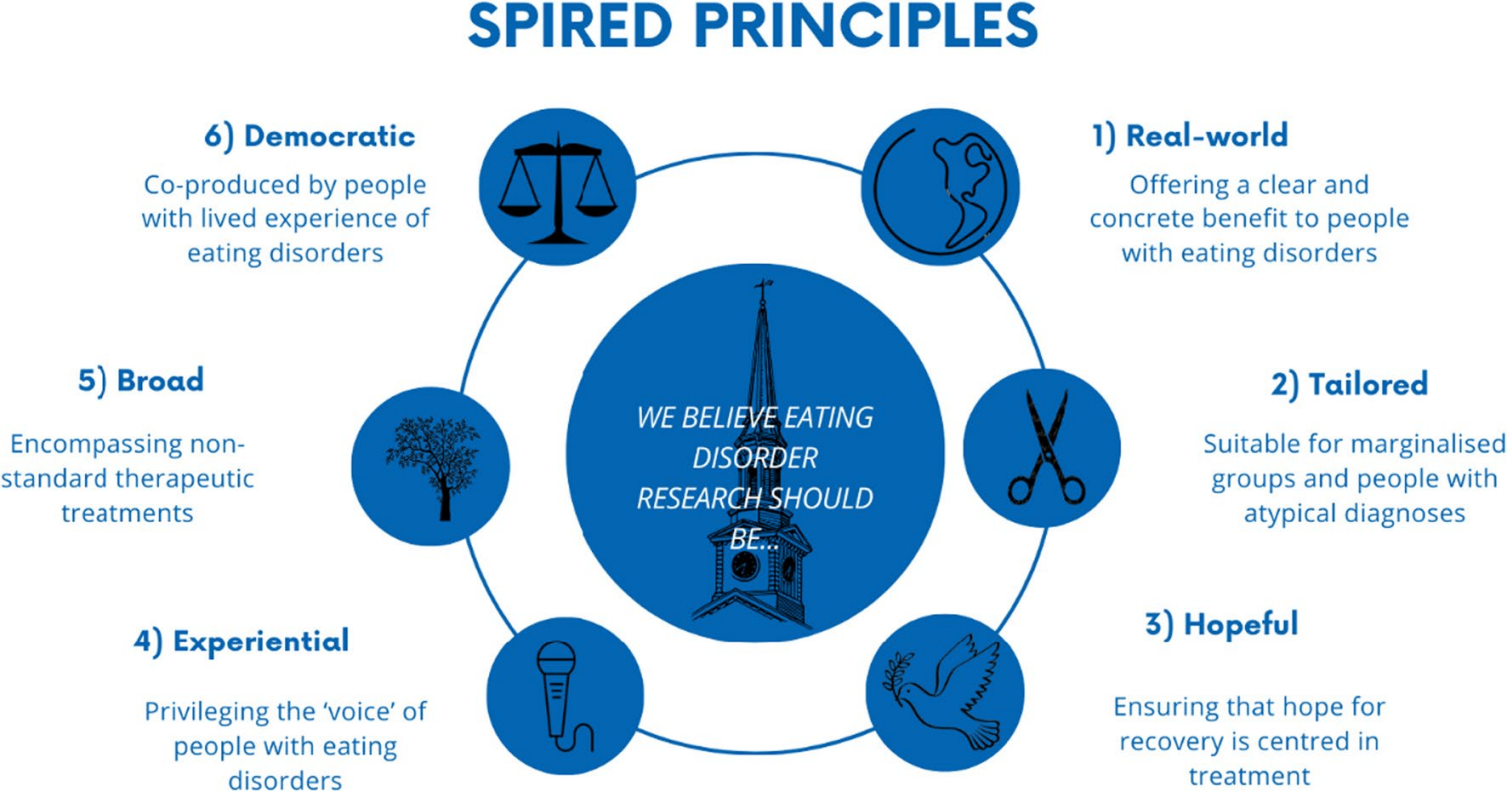
Overview of SPIRED principles

As a result of the process outlined above, the following six principles were determined to be fundamental in guiding our research. We believe in eating disorder research which is: real world, tailored, hopeful, experiential, broad and democratic (see Fig. 1).

We have chosen in this section to integrate the ‘results’ of our decision-making with existing literature, as this best reflects both our aims in doing this work and the process through which the principles came about. Traditional qualitative research results focus solely on ‘participant data’, viewing people experiences, viewpoints, and theorizing as data-points to be analysed by a researcher, and then contextualized within the discussion section of a paper. However, our lived experience panel (along with other clinic members) are not participants, but researchers, and drew on both their own individual experiences but also their understanding and critical assessment of the research literature during this process. This demonstrates the capacity of lived-experience led research to create new forms of knowing outside traditional research binaries, such as those between researcher and researched [63]. Because our discussions as a group, both in the meetings, and over email, involved sharing and reflecting on research findings, this structure has been mirrored in our write-up. The point of this work was to create a set of principles that would be foundational for our work together and could be shared with others. Although this paper describes the process by which we developed these principles, we consider the below results section not the output of a novel co-production process, but also an argument for change [16].

### Real-world

Given the paucity of research on efficacy and acceptability of ED treatments, there is an urgent need for research which is ‘real world’ and makes an immediate, practical impact on the lives of people with eating disorders. Within SPIRED, this could include developing and evaluating novel interventions for eating disorders [93], evaluating, improving and developing ED service provision [109] or engaging with policymaking to ensure it is evidence-based.

Current research does not seem to consistently meet the needs of those with EDs. Although recent research has established connections between the presence of an ED and brain structure and functioning [42, 117], translating findings on neurofeedback and biofeedback into effective treatment has proven difficult, with current therapeutic options proving too invasive and costly [126].

Due to the high mortality rate of EDs and current lack of evidence-based treatment, we call for urgent prioritisation of funding for well-targeted research that can be translated into concrete and immediate changes in service provision [55].

### Tailored

Where evidence-based treatments do exist, researchers have often been slow to adapt them effectively for marginalised groups [2]. We believe that eating disorder treatments and services should adopt an intersectional approach [25], recognising that people have differing identities, such as race, class, gender and sexuality, which interact and result in differing experiences of oppression [36]. Consequently, ED research should offer evidence-based ways of tailoring existing interventions to include minority groups and acknowledge the systemic factors which may create inequalities in accessing ED treatment [17].

Within ED research, particular groups have been excluded from treatment development, and treatment and service provision, so are often missing in the dialogue within eating disorder research. These include: people with other comorbid mental health issues [10, 17, 69, 137], autistic spectrum conditions [3, 22, 40, 62, 118, 121], people with binge-eating disorder, EDNOS/OSFED or ARFID [11, 13, 106], men [103, 112, 120, 134, 138, 140], racially minoritized groups [1, 20, 52, 53, 77, 114, 119, 139] and LGBTIA people [8, 27, 51, 80, 85, 95, 98].

### Hopeful

Whilst acknowledging the issues within current ED research is important, it is vital to hold an optimistic and forward-thinking stance on treatment for EDs. Individuals with eating disorders often experience high levels of hopelessness [116], and as a result interventions which target “hope” and forward looking thinking have been found to be effective in facilitating recovery [64, 116]. Empathy and the provision of hope have been repeatedly found to be the most important features in health professionals working with patients with eating disorders [43, 46, 65, 135].

Evidence suggests that peer support can provide the support and connection necessary to increase people’s sense of hope for recovery, as a key mechanism of change [29, 48, 86, 101, 110].

Interventions which harness peer support have included online discussion forums [67, 86], formal mentoring programmes [57, 99, 100] and clinicians use of their own personal recovery in the treatment of eating disorders [34, 131].

### Experiential

Eating disorder research often focuses on quantitative outcomes such as BMI to evaluate interventions [12, 24]. However solely focusing on BMI as an indicator of recovery is not recommended by NICE guidelines [90]. This narrow focus on BMI can cause iatrogenic harm to patients [88], and marginalise patients with diagnoses aside from AN [130].

The importance placed on measuring people with eating disorders bodies, over listening to their experience, can be understood utilizing the philosophical concept of epistemic injustice; whereby patients’ knowledge about their own condition is disregarded, and they are reduced to passive ‘objects’ to be measured, instead of active and knowing subjects [37].

A significant part of recovery from eating disorders can involve developing a “recovery” voice separate from the illness [18], with effective therapeutic interventions utilising creative ways of developing this [59, 60]. The current focus on BMI as an indicator of recovery does not take patients own knowledge and understanding of their illness into account and overlooks the subjective experiences of those with EDs receiving treatment.

We feel there is a need for an increase in qualitative research which explores the perspectives of ED sufferers and aims to understand their experiences. This may be key for developing more effective interventions in this area [35].

### Broad

Although traditional therapies for EDs can form an important part of ED recovery, they do not address all the factors which have the capacity to have a positive impact of the lives of those with EDs. It is well established in the literature that carers play an important role in recovery [108, 123], as well as peers [57]. Despite acknowledgment that carers might struggle in these roles [33], research exploring which factors impact carer wellbeing, and what support they might benefit from, is still a comparatively new field [122].

There is an emerging evidence base on the impact of holistic therapies on EDs, including art therapy [47, 61], dance [71] and comedy [78]. This is consistent with an understanding that ED recovery involves rebuilding a life outside the ED [135] beyond simply managing the illness [39, 74, 111].

We believe that it is important to broaden out the research focus from the development and evaluation of therapies, to consider provision of support to all parts of the ‘system’ (family members, carers, peers, clinicians) as well as focusing on the development of novel and creative interventions.

### Democratic

Despite the importance of involving patients and carers in eating disorder research and service provision there is often limited ‘co-production in this area’ [87], with inclusion of lived-experience perspectives “scarce to date” [84].

It is our belief that many of the current issues we have highlighted in eating disorder research are the result of not including the perspectives and expertise of people who have experience of eating disorders. Co-production in eating disorder research is particularly important in determining how to define and evaluate recovery in eating disorders (moving away from narrow outcomes like BMI) [68, 125] and towards a richer understanding of the recovery process [135].

We advocate an approach to research that involves service-users/survivors and clinicians working in partnership with other researchers, in all aspects of eating disorder research. This could include setting research priorities, designing research projects, conducting all aspects of research, and disseminating research and engaging the public.

## Discussion

We wanted to trial a ‘principles-first’ approach in developing research projects within our eating disorder research clinic (SPIRED), as we hoped that it would give us a direction and mandate for our work stemming from all members of the clinic. Through a series of workshops, we co-produced a set of principles to guide the direction and process of our work together, agreeing that the eating disorder research we conducted would be real world, tailored, hopeful, experiential, broad, and democratic.

In this section we first offer some reflections on the process from clinic members, highlighting strengths and weaknesses of the approach we took, before setting these in the context of the broader literature.

### Reflections

Members of the SPIRED research clinic wrote reflections on their experience of co-producing principles to guide our research and collaborating on this paper. We present these in their individuality and diversity, instead of coming to a single reflexive position (as authors) on the strengths and limitations of the process we took.

There was consensus that any division between members of the lived experience panel and other researchers and clinicians was not only artificial (as members had a multiplicity of roles and identities in their lives), but also undermined the democratic way we aimed to work together. People are identified here by whether they are a professional member or a lived experience panel member within the SPIRED clinic, however we wanted to emphasize that they will have other identities and areas of expertise. We identified people in this way as we thought it was important to acknowledge the power imbalances that exist between lived experience panel members, and those employed by the trust in a more permanent capacity.

*“[Contrasting lived experience perspectives with clinicians and researchers] creates an artificial binary where our ED defines us—instead of recognising multiple identities. I fit into each of these 'categories’, but the research process runs the risk of reinforcing power differentials and that we ARE the ED. An ED can silence our sense of self and other aspects of identity—the research process shouldn't replay this.”*

-Lived Experience Panel Member

*"I was looking forward to contributing as someone with Lived Experience, because I work as a research student in my day job, so I wear two hats and have seen first-hand the value in incorporating Lived Experience perspectives.”*

-Lived Experience Panel Member

*“As a clinician and researcher who has also had experiences of being on the other side of service provision, I was acutely aware of the ‘us and them’ divisions that play out in both clinical and research settings, and how these divisions can make it harder for people to open up and express fully their views about any care they might or might not have received. We are then missing important information around how to develop interventions and improve care delivery.”*

-Professional Member

One of the most significant things about the process for many SPIRED members was that the initial meeting was one of the first times they were able to be open about, integrate and use their multiple identities. This meeting was felt by many as cathartic, hopeful and powerful, as well as providing a solid foundation for collaboration.

*“As a mental health professional, who was sharing my experiences of being a service user for the first time in front of other professionals, I felt scared and shy at first. Everyone was supportive and reassuring, and I never felt “less than” for sharing my lived experience.”*

-Lived Experience Panel Membe﻿r

*“I was keen that the LEAP was a place that welcomed all perspectives and we put a lot of thought into the initial meetings to create a psychologically safe arena for people to know they could be open and honest. An example of this was me sharing that I had not had good experiences of service use, to help others feel that all perspectives were welcome and to counter the us and them divide.”*

-Professional Member

*“There was such a really open atmosphere in that first meeting—I shared things about my mental illness for the first time in a work context. I think this was such a respectful and trusting basis for the later consensus-based decision-making work we did together in developing the principles.”*

-Professional Member

Part of why the process of co-producing principles was felt to be successful, was because everyone shared similar motivations for wanting to be part of the SPIRED research clinic. These included a motivation to improve eating disorder treatment, alongside a commitment to genuine co-production, as opposed to lived experience involvement just having a ‘rubber-stamping’ function.

*“Having been denied help because of a “normal BMI”, deteriorating and then being on a waiting list for months I felt the need to do something- just anything to take this disappointment and anger towards services and turn it into something a bit positive.”*

-Lived Experience Panel Member

*“I think my main motivations for helping out are guilt, being bluntly honest. I had amazing treatment—the best. Not everyone is so lucky. So, I want to pay that forward and try in any meagre way to give back.”*

-Lived Experience Panel Member

*“I really wanted to work at SPIRED because the people who set it up were so clear about how much they valued co-production as core to the way they worked.”*

-Professional Member

*“I was happy that everyone seemed to be on the same page and have a shared passion for improving lives for people with eating disorders.”*

-Lived Experience Panel Membe﻿r

All the co-authors of this paper except two, were present for the initial meeting and subsequent discussions. However even those who joined the process later, felt aligned with what had already been done.

*“I have been struck by the paucity of research focused on ED interventions, particularly the lack of research which is co-produced with those with lived experience. Despite not being involved in the discussions in which SPIRED principles emerged, I believe the research priorities outlined by SPIRED are incredibly important and reflective of my own values.”*

-Professional Member

*“As I was reading through, I couldn’t believe how aligned they were with my own personal views on co-production and the importance of lived experience perspectives in research […]. This is such a hopeful piece of work.”*

-Professional Member

Those responsible for facilitating the initial meeting, and supporting the lived experience panel going forward, were keen to reduce the burden on them from engaging in this work.

*“I was also aware that speaking about your own difficult experiences can be exhausting, triggering and recovery is not neat; people can relapse and struggle. I didn’t want there to be only a couple of people who were relied on excessively for contributions, so there was an idea that people could dip in and out depending on capacity and interest.”*

-Professional Member

However, despite the shared wish to make lived experience work core to the SPIRED clinic, structural inequalities between the clinic members were a significant barrier to democratic working. Clinicians and researchers were employed on a substantive basis, whereas the lived experience panel were employed on a casualized basis, occasionally doing work—but with limited funding. This meant that often they were not able to lead on aspects of the research.

*“Although I do have lived experience of mental illness (including disordered eating) I’m not one of the Lived Experience Panel members for SPIRED, and it should have been one or many of them that wrote up this paper—which they would have been much better placed to do than me. However sadly we didn’t have a permanently employed lived experience researcher, or enough in our PPI budget to pay for this work to be done on a casualised basis.”*

-Professional Member

Some people felt that this led to differences between how lived experience panel members and other SPIRED clinic members were treated, though others didn’t share this view.

*“[There was a] 'distance' between the researchers and those assigned a 'lived experience' identity in having limited communications from the project.”*

-Lived Experience Panel Member

*"Every communication I had from the PPI team felt like they were talking to an equal member of the team. Same for the paper- I didn’t feel like my thoughts and comments counted less because I was a LEAP member and not a researcher."*

-Lived Experience Panel Member

In general, people felt pride in what we’d achieved together and positive about the personal benefits of creating this paper.

*“I remember feeling very proud of having contributed on that piece of collective work.”*

-Lived Experience Panel Member

*“Lived Experience contributors don't often have a "career pathway" or professional development opportunities, so helping to shape an academic paper—and this was before I had published anything in my own field, as a student—was a really valuable experience.”*

-Lived Experience Panel Member

### Our work in context

We set-up our lived experience led research clinic due to a belief that ED research was not currently adequately meeting the needs of the population it aimed to serve.

It is our view current issues in ED research are partially due to the distinct absence of the voices of those with lived experience of EDs and a lack of co-production in ED research.

We wanted to address the absence or superficiality of co-production in ED research by co-producing a set of principles which would be a mandate for our work together and ensure that it was grounded in what we cared about as a team. Although it initially seemed unlikely that a consensus-based decision-making methodology [56] would be successful, given the range of people with different backgrounds and experiences and priorities in our initial clinical meeting, this has not been our experience. Even the initial questionnaires that were completed by all clinic members*—*prior to meeting*—s*howed significant overlap in which principles people wanted to prioritize, and discussions further solidified these shared understandings and commitment. There was debate at all stages of the process of developing the principles, from initial workshops through to comments on drafts of the paper. These issues were resolved through discussion (during meetings as well as via email),this resolution tended to occur by describing the principles in a nuanced way that incorporated critical feedback from clinic members. All members enthusiastically agreed with the way the principles were framed in the final draft of this paper.

We were fortunate as a research clinic in having funding allocated to us for about 60 h of PPI time annually, as well as a dedicated PPI co-ordinator for our clinic, and a broader PPI team within our NHS trust who were able to advise on issues. This enabled us to take a longer-term strategy for PPI, as opposed to only having funding tied to specific research projects*—*where the aims and the method of the project had already been determined. Even though UK funders often include a requirement for PPI to have occurred at the project development stage [91], meaning that research institutions often have funding available for this specific purpose, it is rarer that PPI funding is allocated to a research team to spend in any way they think appropriate. However, this was essential to the kind of approach we were able to take. Additional funding would have allowed us time and research capacity to use a wider variety of methodological approaches to co-produce principles,we would love to see the work outlined in this paper built on and added to by other researchers trialling different and more systematic approaches for doing so. We believe that it is important to have more substantive lived-experience positions in research, something that we would like to see in our research clinic.

We recruited our PPI panel from social media, as well as patients who were at the point of discharge. As outlined above, POC are not adequately represented on our lived experience advisory panel, something that is a significant issue with many cases of co-production and PPI within research [49]. Developing ongoing collaborations with organisations focused on health research specific to POC, as well as putting systems in place to enhance recruitment of POC to senior positions within research teams [45] are both strategies that could improve these issues. However, neither of these constitutes a ‘quick fix’, and we were keen to work to address the underlying issue causing under-presentation of POC on our LEAP, as opposed to just advertising specifically for more POC which we felt was tokenistic.

We wanted to ensure that the research funding that we applied for corresponded to our research agenda*—*which was derived directly from our principles. However, in trying to develop competitive funding applications which fell under the ‘broad’ principle (i.e., developing and evaluating non-standard therapeutic treatments, including peer-support) it became clear that fewer health research funding sources would consider these kinds of treatments, as opposed to more traditional therapies. It appears that currently, ED research priorities can be guided by funders [38], rather than those with lived experience of EDs, who can provide valuable insight into where research efforts need to be urgently directed.

Priorities and principles and developed as a result of co-production approaches such as that one taken in this paper are often critical in approach and conflict with existing recommendations for eating disorder treatments [89], as approaches which seek to elevate the voices of service users have found in other areas [129]. However, we feel that this conflict has arisen because of the historic marginalization of lived experience voices within mental health research, and the lack of power people with lived experience still have in collaborating on research projects [73]. Given the evidence that research priorities determined by ED service-users produce better interventions and outcomes [132], and the ineffectiveness of current ED treatments [58], we believe it time for a new approach—led by those with lived-experience of eating disorders.

## Conclusions

To our knowledge, we are the first health research group to have co-produced principles as the basis for our work together—putting us more in line with grassroots campaigning organisations [81, 82]. However, these historic organizations rarely outline the process by which they produce their manifestos, which makes it hard to replicated and develop their methodology. There are several limitations to this piece of work, however we hope that other researchers will both build upon and refine the approach outlined in this paper and develop new methodologies for enhancing co-production within health research through the creation of principles to guide their collaborative work. More broadly, we wanted to provide an illustrative case for the value and generative potential of co-producing principles to guide health research and enable long-term collaboration between people with lived experience of an illness, and other researchers [127].

Following the development of SPIRED principles they were included in an All-Party parliamentary group report on eating disorders led by BEAT [16], as an example of how "a relatively small investment can make a big impact in building local research capacity and supporting the application of research findings into clinical practice."

## Data Availability

Not applicable.

## Abbreviations

AN: Anorexia nervosa
BN: Bulimia nervosa
ED: Eating disorder
PPI: Patient and public involvement (in research)
SPFT: Sussex Partnership NHS Foundation Trust

## References

[1] Abood DA, Chandler SB (1997). Race and the role of weight, weight change, and body dissatisfaction in eating disorders. Am J Health Behav.

[2] Acle A, Cook BJ, Siegfried N, Beasley T (2021). Cultural considerations in the treatment of eating disorders among racial/ethnic minorities: a systematic review. J Cross Cult Psychol.

[3] Adamson J, Kinnaird E, Glennon D, Oakley M, Tchanturia K (2020). Carers’ views on autism and eating disorders comorbidity: qualitative study. BJPsych Open.

[4] Adlam J. Shared work and existential engagement in the therapeutic milieu. (2016)

[5] Adlam J. ‘Project Antigone’: a psychosocial exploration of the dynamics of food refusal and force feeding (2014). Doi: 10.13140/2.1.4963.2328

[6] Allen KL, Mountford VA, Elwyn R, Flynn M, Fursland A, Obeid N, Partida G, Richards K, Schmidt U, Serpell L, Silverstein S, Wade T (2023). A framework for conceptualising early intervention for eating disorders. Eur Eat Disord Rev.

[7] Allen KL, Mountford V, Brown A, Richards K, Grant N, Austin A, Glennon D, Schmidt U (2020). First episode rapid early intervention for eating disorders (FREED): from research to routine clinical practice. Early Interv Psychiatry.

[8] Amianto F (2016). Homosexuality and anorexia nervosa: an explorative study on personality traits. Acta Psychopathol.

[9] Aouad P, Hambleton A, Marks P, Maloney D, Calvert S, Caldwell B, McLean SA, Shelton B, Cowan K, Feneley J, Pepin G, Paxton S, Williams M, Meddick T, Squire S, Hickie I, Kay Lambkin F, Touyz S, Maguire S (2022). Setting the top 10 eating disorder research and translation priorities for Australia. Aust N Z J Psychiatry.

[10] Araujo DMR, da Santos GF, Nardi AE (2010). Binge eating disorder and depression: a systematic review. World J Biol Psychiatry.

[11] Arcelus J, Mitchell AJ, Wales J, Nielsen S (2011). Mortality rates in patients with anorexia nervosa and other eating disorders: a meta-analysis of 36 studies. Arch Gen Psychiatry.

[12] Atwood ME, Friedman A (2020). A systematic review of enhanced cognitive behavioral therapy (CBT-E) for eating disorders. Int J Eat Disord.

[13] Bailey AP, Parker AG, Colautti LA, Hart LM, Liu P, Hetrick SE (2014). Mapping the evidence for the prevention and treatment of eating disorders in young people. J Eat Disord.

[14] Beale J (2019). Scientism and scientific imperialism. Int J Philos Stud.

[15] BEAT. Types of Eating Disorder—Beat (2020). https://www.beateatingdisorders.org.uk/types

[16] BEAT. All-Party Parliamentary Group (APPG) on Eating Disorders (2021). Beat. https://www.beateatingdisorders.org.uk/about-beat/policy-work/all-party-parliamentary-group-appg-on-eating-disorders/

[17] Becker AE, Eddy KT, Perloe A (2009). Clarifying criteria for cognitive signs and symptoms for eating disorders in DSM-V. Int J Eat Disord.

[18] Bell NJ (2013). Rhythm and semiotic structures of long-term ambivalence in the dialogical self: eating disorder and recovery voices. J Constr Psychol.

[19] Blackburn J, Minogue V (2014). Developing an eating disorder pathway: a case study. J Ment Health Train Educ Pract.

[20] Bodell LP, Wildes JE, Cheng Y, Goldschmidt AB, Keenan K, Hipwell AE, Stepp SD (2018). Associations between race and eating disorder symptom trajectories in black and white girls. J Abnorm Child Psychol.

[21] Boyle D, Harris M (2009). The challenge of co-production. Lond New Econ Found.

[22] Brede J, Babb C, Jones C, Elliott M, Zanker C, Tchanturia K, Serpell L, Fox J, Mandy W (2020). “For me, the anorexia is Just a symptom, and the cause is the autism”: investigating restrictive eating disorders in autistic women. J Autism Dev Disord.

[23] Brownley KA, Berkman ND, Sedway JA, Lohr KN, Bulik CM (2007). Binge eating disorder treatment: a systematic review of randomized controlled trials. Int J Eat Disord.

[24] Bulik CM, Berkman ND, Brownley KA, Sedway JA, Lohr KN (2007). Anorexia nervosa treatment: a systematic review of randomized controlled trials. Int J Eat Disord.

[25] Burke NL, Schaefer LM, Hazzard VM, Rodgers RF (2020). Where identities converge: the importance of intersectionality in eating disorders research. Int J Eat Disord.

[26] Cahn ES. No more throw-away people: the co-production imperative. Edgar Cahn (2000)

[27] Calzo JP, Austin SB, Micali N (2018). Sexual orientation disparities in eating disorder symptoms among adolescent boys and girls in the UK. Eur Child Adolesc Psychiatry.

[28] CAPO. Introduction, manifesto, demands (n.d.). https://tonybaldwinson.files.wordpress.com/2018/07/undated-campaign-against-psychiatric-oppression-capo.pdf

[29] Cardi V, Ambwani S, Crosby R, Macdonald P, Todd G, Park J, Moss S, Schmidt U, Treasure J (2015). Self-help and recovery guide for eating disorders (SHARED): study protocol for a randomized controlled trial. Trials.

[30] Carel H, Kidd IJ. Epistemic injustice in medicine and healthcare. In: Kidd IJ, Medina J, Pohlhaus G Jr, editors. The Routledge handbook of epistemic injustice. Routledge; 2017. p. 336–46.

[31] Christopher S, Saha R, Lachapelle P, Jennings D, Colclough Y, Cooper C, Cummins C, Eggers MJ, FourStar K, Harris K, Kuntz SW, LaFromboise V, LaVeaux D, McDonald T, Bird JR, Rink E, Webster L (2011). Applying indigenous community-based participatory research principles to partnership development in health disparities research. Fam Commun Health.

[32] Clark M (2015). Co-production in mental health care. Ment Health Rev J.

[33] Coomber K, King RM (2012). Coping strategies and social support as predictors and mediators of eating disorder carer burden and psychological distress. Soc Psychiatry Psychiatr Epidemiol.

[34] Costin C (2002). Been there, done that: clinicians’ use of personal recovery in the treatment of eating disorders. Eat Disord.

[35] Craig P, Dieppe P, Macintyre S, Michie S, Nazareth I, Petticrew M (2013). Developing and evaluating complex interventions: The new medical research council guidance. Int J Nurs Stud.

[36] Crenshaw K. Demarginalizing the intersection of race and sex: a Black feminist critique of antidiscrimination doctrine, feminist theory, and antiracist politics [1989]. In: Bartlett K, editor. Feminist legal theory. Routledge; 2018. p. 57–80.

[37] Crichton P, Carel H, Kidd IJ (2017). Epistemic injustice in psychiatry. BJPsych Bull.

[38] Davies H. Eating disorders bear the brunt of the paucity in mental health research funding. The Psych PhD Pathway. 2020. https://thepsychphdpathway.com/2020/09/15/eating-disorders-research-funding/.

[39] Dawson L, Rhodes P, Touyz S (2014). “Doing the impossible”: the process of recovery from chronic anorexia nervosa. Qual Health Res.

[40] Dell’Osso L, Carpita B, Gesi C, Cremone IM, Corsi M, Massimetti E, Muti D, Calderani E, Castellini G, Luciano M, Ricca V, Carmassi C, Maj M (2018). Subthreshold autism spectrum disorder in patients with eating disorders. Compr Psychiatry.

[41] Department of Health. Liberating the NHS: No decision about me, without me. 2012.

[42] Donnelly B, Touyz S, Hay P, Burton A, Russell J, Caterson I (2018). Neuroimaging in bulimia nervosa and binge eating disorder: a systematic review. J Eat Disord.

[43] Evans EJ, Hay PJ, Mond J, Paxton SJ, Quirk F, Rodgers B, Jhajj AK, Sawoniewska MA (2011). Barriers to help-seeking in young women with eating disorders: a qualitative exploration in a longitudinal community survey. Eat Disord.

[44] Faulkner A (2017). Survivor research and mad studies: the role and value of experiential knowledge in mental health research. Disabil Soc.

[45] Faulkner A, Thompson R (2021). Uncovering the emotional labour of involvement and co-production in mental health research. Disabil Soc.

[46] Fogarty S, Ramjan LM (2016). Factors impacting treatment and recovery in anorexia nervosa: qualitative findings from an online questionnaire. J Eat Disord.

[47] Frisch MJ, Franko DL, Herzog DB (2006). Arts-based therapies in the treatment of eating disorders. Eat Disord.

[48] Gallagher MW, Long LJ, Richardson A, D’Souza J, Boswell JF, Farchione TJ, Barlow DH (2020). Examining hope as a transdiagnostic mechanism of change across anxiety disorders and CBT treatment protocols. Behav Ther.

[49] Gibbins KJ, Lo JO (2022). What matters to whom: patient and public involvement in research. Clin Obstet Gynecol.

[50] Gillard S, Foster R, Gibson S, Goldsmith L, Marks J, White S (2017). Describing a principles-based approach to developing and evaluating peer worker roles as peer support moves into mainstream mental health services. Ment Health Soc Incl.

[51] Goldhammer HB, Maston ED, Keuroghlian AS (2019). Addressing eating disorders and body dissatisfaction in sexual and gender minority youth. Am J Prev Med.

[52] Gordon KH, Brattole MM, Wingate LR, Joiner TE (2006). The impact of client race on clinician detection of eating disorders. Behav Ther.

[53] Gordon KH, Perez M, Joiner TE (2002). The impact of racial stereotypes on eating disorder recognition. Int J Eat Disord.

[54] Grattidge L, Purton T, Auckland S, Lees D, Mond J (2021). Participatory action research in suicide prevention program evaluation: opportunities and challenges from the National Suicide Prevention Trial, Tasmania. Aust N Z J Public Health.

[55] Great Britain & Parliamentary and Health Service Ombudsman. Ignoring the alarms: how NHS eating disorder services are failing patients. 2017.

[56] Guinaudie C, Mireault C, Tan J, Pelling Y, Jalali S, Malla A, Iyer SN (2020). Shared decision making in a youth mental health service design and research project: insights from the pan-canadian access open minds network. Patient Patient-Center Outcomes Res.

[57] Hanly F, Torrens-Witherow B, Warren N, Castle D, Phillipou A, Beveridge J, Jenkins Z, Newton R, Brennan L (2020). Peer mentoring for individuals with an eating disorder: a qualitative evaluation of a pilot program. J Eat Disord.

[58] Hay P (2013). A systematic review of evidence for psychological treatments in eating disorders: 2005–2012. Int J Eat Disord.

[59] Hilliard RE (2001). The use of cognitive-behavioral music therapy in the treatment of women with eating disorders. Music Ther Perspect.

[60] Hodge L, Simpson S (2016). Speaking the unspeakable: artistic expression in eating disorder research and schema therapy. Arts Psychother.

[61] Holmqvist G, Persson CL (2012). Is there evidence for the use of art therapy in treatment of psychosomatic disorders, eating disorders and crisis? A comparative study of two different systems for evaluation. Scand J Psychol.

[62] Huke V, Turk J, Saeidi S, Kent A, Morgan JF (2013). Autism spectrum disorders in eating disorder populations: a systematic review. Eur Eat Disord Rev.

[63] Ingram RA (2016). Doing mad studies: making (non)sense together. Intersect Glob J Soc Work Anal Res Polity Pract.

[64] Irving LM, Cannon R, Snyder CR (2000). Chapter 14 - starving for hope: goals, agency, and pathways in the development and treatment of eating disorders. Handbook of hope.

[65] Jewell T, Blessitt E, Stewart C, Simic M, Eisler I (2016). Family therapy for child and adolescent eating disorders: a critical review. Fam Process.

[66] Karnieli-Miller O, Strier R, Pessach L (2009). Power relations in qualitative research. Qual Health Res.

[67] Kendal S, Kirk S, Elvey R, Catchpole R, Pryjmachuk S (2017). How a moderated online discussion forum facilitates support for young people with eating disorders. Health Expect.

[68] Kenny TE, Lewis SP (2021). reconceptualizing recovery: integrating lived experience perspectives into traditional eating disorder recovery frameworks. Psychiatr Serv.

[69] Keski-Rahkonen A, Mustelin L (2016). Epidemiology of eating disorders in Europe: prevalence, incidence, comorbidity, course, consequences, and risk factors. Curr Opin Psychiatry.

[70] King AJ, Brophy LM, Fortune TL, Byrne L (2020). factors affecting mental health professionals’ sharing of their lived experience in the workplace: a scoping review. Psychiatr Serv.

[71] Krantz AM (1999). Growing into her body: dance/movement therapy for women with eating disorders. Am J Dance Ther.

[72] LaMarre A, Levine MP, Holmes S, Malson H (2022). An open invitation to productive conversations about feminism and the spectrum of eating disorders (part 1): basic principles of feminist approaches. J Eat Disord.

[73] Lambert N, Carr S (2018). ‘Outside the original remit’: co-production in UK mental health research, lessons from the field. Int J Ment Health Nurs.

[74] Lamoureux M, Bottorff J (2005). “Becoming the real me”: recovering from anorexia nervosa. Health Care Women Int.

[75] Lewis HK, Foye U. From prevention to peer support: a systematic review exploring the involvement of lived-experience in eating disorder interventions. Ment Health Rev J. 2021.

[76] Lindvall Dahlgren C, Wisting L, Rø Ø (2017). Feeding and eating disorders in the DSM-5 era: a systematic review of prevalence rates in non-clinical male and female samples. J Eat Disord.

[77] Lynch SL. Eating disorders in African American women: Incorporating race into considerations of etiology and treatment [Psy.D., Widener University, Institute for Graduate Clinical Psychology]. 2003. https://search.proquest.com/docview/305257106/abstract/3009282B20AE4BC6PQ/1.

[78] MacRury I (2012). Humour as ‘social dreaming’: stand-up comedy as therapeutic performance. Psychoanal Cult Soc.

[79] Madden M, Speed E (2017). Beware zombies and unicorns: toward critical patient and public involvement in health research in a neoliberal context. Front Sociol.

[80] McClain Z, Peebles R (2016). Body image and eating disorders among lesbian, gay, bisexual, and transgender youth. Pediatr Clin.

[81] Mental Patients Union. Declaration of Intent. 1973. https://libcom.org/article/mental-patients-union-1973.

[82] MPU. The Need for a Mental Patients’ Union. Some Proposals. 1976.

[83] Murray SB, Pila E, Griffiths S, Le Grange D (2017). When illness severity and research dollars do not align: are we overlooking eating disorders?. World Psychiatry.

[84] Musić S, Elwyn R, Fountas G, Gnatt I, Jenkins ZM, Malcolm A, Miles S, Neill E, Simpson T, Yolland CO, Phillipou A (2021). Valuing the voice of lived experience of eating disorders in the research process: benefits and considerations. Aust N Z J Psychiatry.

[85] Nagata JM, Ganson KT, Austin SB (2020). Emerging trends in eating disorders among sexual and gender minorities. Curr Opin Psychiatry.

[86] Naslund JA, Aschbrenner KA, Marsch LA, Bartels SJ (2016). The future of mental health care: peer-to-peer support and social media. Epidemiol Psychiatr Sci.

[87] Newton T (2001). Consumer involvement in the appraisal of treatments for people with eating disorders: a neglected area of research?. Eur Eat Disord Rev.

[88] NHS England. Adult eating disorders: community, inpatient and intensive day patient care: guidance for commissioners and providers, 28 (2019).

[89] NICE. Eating disorders: Recognition and treatment (2017a).

[90] NICE. Overview | Eating disorders: recognition and treatment | Guidance | NICE. (2017b). NICE. https://www.nice.org.uk/guidance/ng69.

[91] NIHR. A brief guide to public involvement in funding applications. 2020. https://www.nihr.ac.uk/documents/a-brief-guide-to-public-involvement-in-funding-applications/24162.

[92] Obeid N, McVey G, Seale E, Preskow W, Norris ML (2020). Cocreating research priorities for anorexia nervosa: the Canadian eating disorder priority setting partnership. Int J Eat Disord.

[93] Oldershaw A, Lavender T, Basra R, Startup H (2022). SPEAKS study: study protocol of a multisite feasibility trial of the Specialist Psychotherapy with Emotion for Anorexia in Kent and Sussex (SPEAKS) intervention for outpatients with anorexia nervosa or otherwise specified feeding and eating disorders, anorexia nervosa type. BMJ Open.

[94] Oliver K, Kothari A, Mays N (2019). The dark side of coproduction: do the costs outweigh the benefits for health research?. Health Res Policy Syst.

[95] Parker LL, Harriger JA (2020). Eating disorders and disordered eating behaviors in the LGBT population: a review of the literature. J Eat Disord.

[96] Peat CM, Berkman ND, Lohr KN, Brownley KA, Bann CM, Cullen K, Quattlebaum MJ, Bulik CM (2017). Comparative effectiveness of treatments for binge-eating disorder: systematic review and network meta-analysis. Eur Eat Disord Rev.

[97] Pilgrim D (2018). Co-production and involuntary psychiatric settings. Ment Health Rev J.

[98] Protos K (2020). Restricting the gendered body: understanding the trans-masculine adolescent with anorexia. Clin Soc Work J.

[99] Purcell J, Lister S, McCormack J, Caswell J, Logie K, Wade S, Stringer M (2014). Reaching out for hope—a peer support program. J Eat Disord.

[100] Ramjan LM, Fogarty S, Nicholls D, Hay P (2018). Instilling hope for a brighter future: a mixed-method mentoring support programme for individuals with and recovered from anorexia nervosa. J Clin Nurs.

[101] Ranzenhofer LM, Wilhelmy M, Hochschild A, Sanzone K, Walsh BT, Attia E (2020). Peer mentorship as an adjunct intervention for the treatment of eating disorders: a pilot randomized trial. Int J Eat Disord.

[102] recoveryinthebin, A. A simple guide to co-production. Recovery in the Bin. 2018. https://recoveryinthebin.org/2018/07/03/a-simple-guide-to-co-production/.

[103] Robinson KJ, Mountford VA, Sperlinger DJ (2013). Being men with eating disorders: perspectives of male eating disorder service-users. J Health Psychol.

[104] Rose D (2017). Service user/survivor-led research in mental health: epistemological possibilities. Disabil Soc.

[105] Rose D, Kalathil J (2019). Power, privilege and knowledge: the untenable promise of co-production in mental “health”. Front Sociol.

[106] Santomauro DF, Melen S, Mitchison D, Vos T, Whiteford H, Ferrari AJ (2021). The hidden burden of eating disorders: an extension of estimates from the Global Burden of Disease Study 2019. Lancet Psychiatry.

[107] Schulz AJ, Israel BA, Selig SM, Bayer IS. Development and implementation of principles for community-based research in public health. In:Research strategies for community practice. Routledge; 1998.

[108] Sepulveda AR, Lopez C, Todd G, Whitaker W, Treasure J (2008). An examination of the impact of “the Maudsley eating disorder collaborative care skills workshops” on the well being of carers: a pilot study. Soc Psychiatry Psychiatr Epidemiol.

[109] Shaw H, Robertson S, Ranceva N (2021). What was the impact of a global pandemic (COVID-19) lockdown period on experiences within an eating disorder service? A service evaluation of the views of patients, parents/carers and staff. J Eat Disord.

[110] Smale K, Pitt J (2013). Eating disorder mentor program. J Eat Disord.

[111] Smethurst L, Kuss D (2018). ‘Learning to live your life again’: an interpretative phenomenological analysis of weblogs documenting the inside experience of recovering from anorexia nervosa. J Health Psychol.

[112] Soban C (2006). What about the boys?: addressing issues of masculinity within male anorexia nervosa in a feminist therapeutic environment. Int J Men’s Health.

[113] Solmi F, Bould H, Lloyd EC, Lewis G (2021). The shrouded visibility of eating disorders research. Lancet Psychiatry.

[114] Sonneville KR, Lipson SK (2018). Disparities in eating disorder diagnosis and treatment according to weight status, race/ethnicity, socioeconomic background, and sex among college students. Int J Eat Disord.

[115] SPFT. Get Involved in Research. Sussex Partnership NHS Foundation Trust. 2023c. https://www.sussexpartnership.nhs.uk/our-research/get-involved-our-research

[116] Stavarski DH, Alexander RK, Ortiz SN, Wasser T (2019). Exploring nurses’ and patients’ perceptions of hope and hope-engendering nurse interventions in an eating disorder facility: a descriptive cross-sectional study. J Psychiatr Ment Health Nurs.

[117] Steward T, Menchon JM, Jiménez-Murcia S, Soriano-Mas C, Fernandez-Aranda F (2018). Neural network alterations across eating disorders: a narrative review of fMRI studies. Curr Neuropharmacol.

[118] Stewart CS, McEwen FS, Konstantellou A, Eisler I, Simic M (2017). Impact of ASD traits on treatment outcomes of eating disorders in girls. Eur Eat Disord Rev.

[119] Striegel-Moore RH, Wilfley DE, Pike KM, Dohm F-A, Fairburn CG (2000). Recurrent binge eating in black American women. Arch Fam Med.

[120] Thapliyal P, Mitchison D, Hay P (2017). Insights into the experiences of treatment for an eating disorder in men: a qualitative study of autobiographies. Behav Sci.

[121] Treasure J (2013). Coherence and other autistic spectrum traits and eating disorders: building from mechanism to treatment. The Birgit Olsson lecture. Nord J Psychiatry.

[122] Treasure J, Nazar BP (2016). Interventions for the carers of patients with eating disorders. Curr Psychiatry Rep.

[123] Treasure J, Todd G, Latzer Y, Stein D (2016). Interpersonal maintaining factors in eating disorder: skill sharing interventions for carers. Bio-psycho-social contributions to understanding eating disorders.

[124] Turnhout E, Metze T, Wyborn C, Klenk N, Louder E (2020). The politics of co-production: participation, power, and transformation. Curr Opin Environ Sustainabil. Curr Opin Environ Sustainabil.

[125] Turton P, Demetriou A, Boland W, Gillard S, Kavuma M, Mezey G, Mountford V, Turner K, White S, Zadeh E, Wright C (2011). One size fits all: or horses for courses? recovery-based care in specialist mental health services. Soc Psychiatry Psychiatr Epidemiol.

[126] Val Laillet D, Aarts E, Weber B, Ferrari M, QuaresimaStoeckel VLE, Alonso-Alonso M, Audette M, Malbert CH, Stice E (2015). Neuroimaging and neuromodulation approaches to study eating behavior and prevent and treat eating disorders and obesity. NeuroImage Clin.

[127] van Furth EF, van der Meer A, Cowan K (2016). Top 10 research priorities for eating disorders. Lancet Psychiatry.

[128] van Hoeken D, Hoek HW (2020). Review of the burden of eating disorders: mortality, disability, costs, quality of life, and family burden. Curr Opin Psychiatry.

[129] Verschuere B, Brandsen T, Pestoff V (2012). Co-production: the state of the art in research and the future agenda. VOLUNTAS Int J Volunt Nonprofit Organ.

[130] Virgo H, NCMH, Stewart C. Dump The Scales Campaign. NCMH. 2019. https://www.ncmh.info/2019/02/25/dump-the-scales/.

[131] de Vos JA, Netten C, Noordenbos G (2016). Recovered eating disorder therapists using their experiential knowledge in therapy: a qualitative examination of the therapists’ and the patients’ view. Eat Disord.

[132] Wade TD, Hart LM, Mitchison D, Hay P (2021). Driving better intervention outcomes in eating disorders: a systematic synthesis of research priority setting and the involvement of consumer input. Eur Eat Disord Rev.

[133] Weist MD, Sander MA, Walrath C, Link B, Nabors L, Adelsheim S, Moore E, Jennings J, Carrillo K (2005). Developing principles for best practice in expanded school mental health. J Youth Adolesc.

[134] Weltzin TE, Weisensel N, Franczyk D, Burnett K, Klitz C, Bean P (2005). Eating disorders in men: update. J Men’s Health Gend.

[135] Wetzler S, Hackmann C, Peryer G, Clayman K, Friedman D, Saffran K, Silver J, Swarbrick M, Magill E, van Furth EF, Pike KM (2020). A framework to conceptualize personal recovery from eating disorders: a systematic review and qualitative meta-synthesis of perspectives from individuals with lived experience. Int J Eat Disord.

[136] Williams O, Robert G, Martin G, Hanna E, O’Hara J (2020). Is Co-production just really good PPI? Making sense of patient and public involvement and co-production networks. Decent Health Care Netw Reshap Org Deliv Healthc.

[137] Woodside BD, Staab R (2006). Management of psychiatric comorbidity in anorexia nervosa and bulimia nervosa. CNS Drugs.

[138] Wooldridge T, Lytle PP (2012). An overview of anorexia nervosa in males. Eat Disord.

[139] Yanovski SZ (2000). Eating disorders, race, and mythology. Arch Fam Med.

[140] Zhang C (2014). What can we learn from the history of male anorexia nervosa?. J Eat Disord.

